# Potential Value of Genomic Copy Number Variations in Schizophrenia

**DOI:** 10.3389/fnmol.2017.00204

**Published:** 2017-06-21

**Authors:** Chuanjun Zhuo, Weihong Hou, Chongguang Lin, Lirong Hu, Jie Li

**Affiliations:** ^1^Department of Psychological Medicine, Wenzhou Seventh People's HospitalWenzhou, China; ^2^Department of Psychological Medicine, Tianjin Anding HospitalTianjin, China; ^3^Department of Biology, University of North Carolina at CharlotteCharlotte, NC, United States; ^4^Department of Biochemistry and Molecular Biology, Zhengzhou UniversityZhengzhou, China

**Keywords:** schizophrenia, copy number variations, single nucleotide polymorphisms, CRISPR/Cas9, neuropsychiatric disorder

## Abstract

Schizophrenia is a devastating neuropsychiatric disorder affecting approximately 1% of the global population, and the disease has imposed a considerable burden on families and society. Although, the exact cause of schizophrenia remains unknown, several lines of scientific evidence have revealed that genetic variants are strongly correlated with the development and early onset of the disease. In fact, the heritability among patients suffering from schizophrenia is as high as 80%. Genomic copy number variations (CNVs) are one of the main forms of genomic variations, ubiquitously occurring in the human genome. An increasing number of studies have shown that CNVs account for population diversity and genetically related diseases, including schizophrenia. The last decade has witnessed rapid advances in the development of novel genomic technologies, which have led to the identification of schizophrenia-associated CNVs, insight into the roles of the affected genes in their intervals in schizophrenia, and successful manipulation of the target CNVs. In this review, we focus on the recent discoveries of important CNVs that are associated with schizophrenia and outline the potential values that the study of CNVs will bring to the areas of schizophrenia research, diagnosis, and therapy. Furthermore, with the help of the novel genetic tool known as the Clustered Regularly Interspaced Short Palindromic Repeats-associated nuclease 9 (CRISPR/Cas9) system, the pathogenic CNVs as genomic defects could be corrected. In conclusion, the recent novel findings of schizophrenia-associated CNVs offer an exciting opportunity for schizophrenia research to decipher the pathological mechanisms underlying the onset and development of schizophrenia as well as to provide potential clinical applications in genetic counseling, diagnosis, and therapy for this complex mental disease.

## Introduction

Schizophrenia is a complex neuropsychiatric disorder typically characterized by symptoms such as delusions and hallucinations, cognitive dysfunction, social deficit, and apathy (Owen et al., 2016). It is estimated that the prevalence of schizophrenia is nearly 1% worldwide, and this devastating mental illness has imposed a considerable burden on both families and society (Hor and Taylor, 2010; Foster et al., 2012; Global Burden of Disease Study, 2015; Owen et al., 2016). To date, the exact etiology of this disease remains unknown. However, it has been recognized that genetics play a pivotal role in the early onset and development of schizophrenia (International Schizophrenia et al., 2009; Chen et al., 2015; Kavanagh et al., 2015; Owen et al., 2016). In fact, the disease is inherited by as many as 80% of patients suffering from schizophrenia (Cardno et al., 1999). In spite of the substantial hereditary component in this mental illness, the complexity underlying the assumption that schizophrenia is a genetic disease has been realized with the following observations as described previously (Crow, 1999): (1) some degree of discordance for schizophrenia between individuals of monozygotic twin pairs in twin studies; (2) sharp risk decrease for individuals beyond the first degree relatives of the schizophrenia patients; (3) unequal disease risk for parents, children, and siblings of the patients with schizophrenia; and (4) the paradox of schizophrenia with the persistence of the disease at relatively high prevalence in spite of a substantial biological/fecundity disadvantage. Therefore, it is becoming clearer that a complex interplay between the genetic and environmental factors may contribute to both the early onset and the development of schizophrenia.

Recent studies into the genetic causes underlying schizophrenia using comparative genomic hybridization, single nucleotide polymorphism (SNP) chips, and gene sequencing have identified different forms of genetic variants such as SNPs and copy number variations (CNVs) that have been shown to be associated with this disease (International Schizophrenia, 2008; St Clair, 2009; Levinson et al., 2011; Grayton et al., 2012; Kirov et al., 2012; Malhotra and Sebat, 2012; Hamshere et al., 2013; Ripke et al., 2013; Purcell et al., 2014). Unlike SNPs, CNVs are defined as intermediate-sized genetic variants, generally with a size of 50 bp to several megabase pairs (Mbp) in length, consist of deletions (Del) or duplications (Dup), and are invisible under microscopy (Girirajan et al., 2011). CNVs can affect one or multiple genes within the region of the CNVs, resulting in profound effects on gene expression and function (Girirajan et al., 2011). Extensive studies have demonstrated that CNVs are ubiquitously distributed in the human genome, with up to 25% of the human genome containing CNVs, and they are responsible for the evolution, population diversity, and different forms of diseases (St Clair, 2009; Girirajan et al., 2011; Malhotra and Sebat, 2012). Recent advancements in genomic technologies, including gene sequencing, genome-wide association studies, and comparative genomic hybridization, have accelerated the identification and characterization of pathogenic CNVs. In particular, the most recent decade has witnessed rapid progress in identifying the number and type of genetic variants conferring the genetic burden of a range of genetically related diseases such as schizophrenia (International Schizophrenia, 2008; St Clair, 2009; Girirajan et al., 2011; Ingason et al., 2011; Grayton et al., 2012; Guha et al., 2013; Mulle et al., 2014; Purcell et al., 2014). The discovery of schizophrenia-associated CNVs also has offered an exciting opportunity for schizophrenia research to decipher the pathological mechanisms concerning the early onset and development of this complex disease as well as to provide potential clinical applications in genetic counseling, diagnosis, and therapy for schizophrenia. In this review, we focus on the recent findings related to the important schizophrenia-associated CNVs and outline the potential values that the study of CNVs could bring to the areas of schizophrenia research, diagnosis, and therapy. Furthermore, we propose that the pathogenic CNVs could be corrected with the novel genetic tool known as the Clustered Regularly Interspaced Short Palindromic Repeats-associated nuclease 9 (CRISPR/Cas9) system. Finally, we describe challenges in the study of schizophrenia-associated CNVs as well as future directions of the potential clinical applications in genetic counseling, diagnosis, and therapy for this complex mental disease.

## Methods

The databases PubMed, Google Scholar, Baidu Scholar, and Wanfang Med Online were searched for relevant literature by using the following keywords: schizophrenia, neuropsychiatric disorder, mental disorder, genetic variants, copy number variations, single nucleotide polymorphisms, gene editing, and CRISPR/Cas9. From the articles identified by the literature search and review, we excluded those with lack of relevance, including the most relevant, original research articles.

## Current major findings and understanding of genomic CNVs in schizophrenia

### Current major findings of important schizophrenia-associated CNVs and affected genes in their intervals

**Figure 1:**
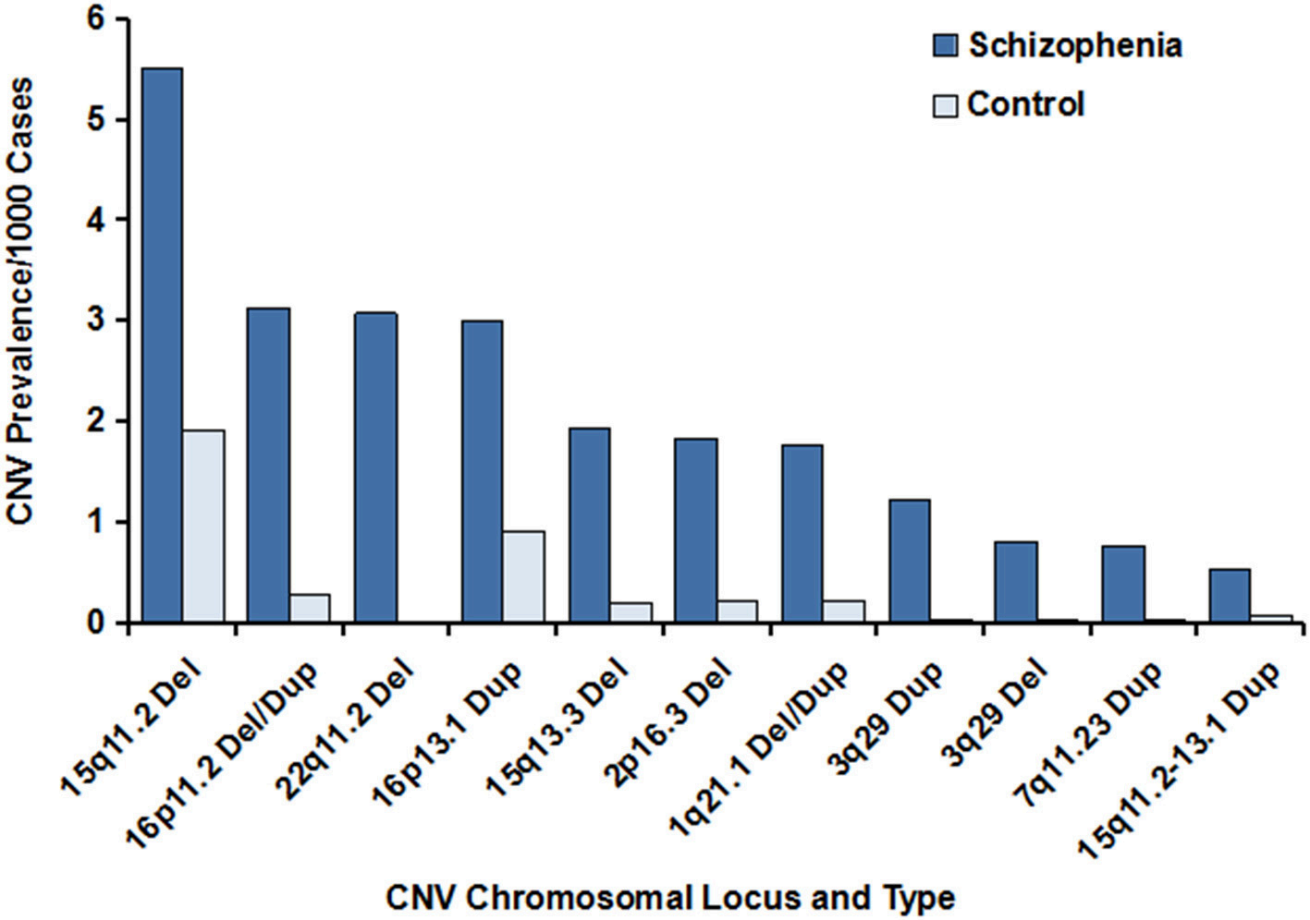
Prevalence of the major CNVs associated with schizophrenia. The data were generated from previous studies, and the references are cited in Table 1. The prevalence of the major CNVs in individuals with schizophrenia vs. controls is illustrated, with the deletion at 15q11.2 being the CNV with the highest frequency. Of note, the prevalence of the deleted schizophrenia-associated CNV at 22q11.2 in the unaffected control individuals was zero. In addition, extremely low frequencies of the CNVs at 3q29 (deletions), 3q29 (duplications), and 7q11.23 (duplications) were observed in controls, compared with schizophrenia cases.

Multiple lines of evidence have indicated the importance of CNVs in schizophrenia. The last decade has witnessed rapid progress in the identification and characterization of schizophrenia-associated CNVs, some of which have been subsequently replicated and validated by independent studies with a large sample size of schizophrenia cases and unaffected controls. They mainly included the CNVs located on chromosomes 1q21.1, 2p16.3, 3q29, 7q11.23, 15q11.2, 15q11.2–13.1, 15q13.3, 16p11.2, 16p13.1, and 22q11.2 (Table 1 and Figure 1). Pathway analysis of the CNVs has shown that the affected genes within the regions of the CNVs are involved in numerous neuronal signaling pathways such as the neuregulin-ErbBand glutamate neurotransmission pathways. One of the mechanisms whereby CNVs may be a causative factor for schizophrenia is by exerting a profound effect on the expression and function of the genes covered by the CNVs. In fact, a large proportion of these CNVs disrupt single or multiple genes, whereas other CNVs may not cover any known genes within their intervals. For example, the schizophrenia-associated CNV deletion at 2p16.3 has been shown to disrupt *NRXN1* gene, a member of the neurexin family, because the promoter and first exon of the *NRXN1* gene fall within the interval of the CNV (Kirov et al., 2008, 2009; Malhotra and Sebat, 2012). The *XRXN1* gene encodes Neurexin1 protein, a presynaptic cell-adhesion molecule involved in regulation of Ca2+-mediated neurotransmitter release at both synapses and neuromuscular junctions in humans. Through interacting with other synaptic proteins, so-called neuroligins (a family with five transmembrane proteins that are mainly located on the postsynaptic surface of synapses), Neurexin 1 participates substantially in the synthesis and release of neurotransmitters at synapses, mediation of signaling, and connection of neurons into communicating networks (Sudhof, 2008; Kirov et al., 2009). Mutations in the *XRXN1* gene have been closely correlated with a high risk of schizophrenia and autism spectrum disorders. These findings have been validated and extended by different research teams using a large sample size of schizophrenia patients and unaffected control individuals, indicating the critical role for the *XRXN1* gene in schizophrenia and establishing the importance of this CNV in schizophrenia (International Schizophrenia, 2008; Mefford et al., 2008; Stefansson et al., 2008; Walsh et al., 2008; Need et al., 2009; Ikeda et al., 2010; Levinson et al., 2011; Rosenfeld et al., 2012; Szatkiewicz et al., 2014). Of the genes located on chromosome 1q21.1 and disrupted by this CNV, the gap junction genes connexin 40 and connexin 50 participate directly in intercellular exchanges of neurotransmitters (e.g., glutamate) in the synaptic transmission of the central nervous system; and a matched case–control study of the two connexin genes found that mutations in connexin 50 were strongly correlated with schizophrenia (Ni et al., 2007; International Schizophrenia, 2008; Levinson et al., 2011). In a cohort of schizophrenia cases and controls of Ashkenazi Jewish descent with CNV microdeletions of more than 500 kb in length on chromosome 3q29, known to confer risk for schizophrenia and other mental illnesses such as intellectual disability and autism spectrum disorder, researchers focused on *de novo* CNV deletions at chromosome 3q29 that were 836 kb in size and found that at least 19 genes within this CNV interval were annotated, of which paralogous to X-linked ID genes and strong candidate risk genes for schizophrenia susceptibility, including Disks large homolog 1 (DLG1) and p21-acivated kinase 2 (PAK2), were revealed. DLG1, also referred to as synapse-associated protein 97 (SAP97), encoded by the SAP97 gene, has been well-documented to bind to neuroligin and glutamate receptor type 1, a member of the glutamate-gated ion channel family, resulting in disturbance of the glutamate neurotransmission system, an important signaling system implicated in the pathology of schizophrenia (Mefford et al., 2008; Zhou et al., 2008; Howard et al., 2010; Ikeda et al., 2010; Mulle et al., 2010; Nash et al., 2010; Yuan et al., 2014). Aside from DLG1, PAK2 is of interest as it has been shown to diminish the inhibitory interaction between Rac1 and RhoGD1; therefore, it is involved in modulation of the synaptic plasticity and dendritic spine morphology (Mulle et al., 2010). The CNV with duplications on chromosome 7q11.2 has been identified to be responsible for Williams-Beuren region duplication syndrome, which is also known as 7q11.2 duplication syndrome. This syndrome is featured by a range of neurodevelopmental and behavioral abnormalities, and additional copies of the two elastin genes responsible for anomalies of connective tissue as well as GTF21 implicated in mental retardation within the intervals are likely to contribute to some of the characteristics observed in 7q11.2 duplication syndrome; however, the exact pathology for this syndrome remains unclear (Kirov et al., 2012; Mulle et al., 2014). The CNVs at chromosome 15q11.2 have been demonstrated to affect the TUBGCP5, CYFIP1, NIPA2, and NIPA1 genes (Ingason et al., 2011; Moreno-De-Luca et al., 2013). A number of studies have reported that the proteins encoded by the NIPA1 and NIPA2 genes are related to Mg^2+^ transportation (Zhao et al., 2013). The CNV duplications on chromosome 15q11.2–15q13.1 have been reported to be associated with schizophrenia and other neurodevelopmental disorders (International Schizophrenia, 2008; Malhotra and Sebat, 2012). Of these genes spanning the duplications of the CNVs, extra copies or dosages of the UBE3A gene are likely to be a candidate gene conferring the neurodevelopmental phenotype and to exert an effect on the glutamate neurotransmission system through the synaptic protein Arc. Studies have shown that the CNVs at 15q13.3 can disrupt the expression and function of the human α7 neuronal nicotinic acetylcholine receptor gene, an important gene highly expressed in the central nervous system and closely correlated to multiple mental disorders with cognitive impairments, including schizophrenia, by mediating rapid signal transmission at synapses (Sinkus et al., 2015). Rare CNVs on 16p11.2 have been implicated in intellectual disability in children, severe early-onset obesity, autism spectrum disorders, and schizophrenia; and they are linked to the developmental delay phenotype (Nielsen et al., 2010; Guha et al., 2013). The genes disturbed by the CNVs mainly involve the SH2B1, TUFM, ATP2A1, and ATP2A2 genes, of which mutations of the single ATP2A2 gene in Darier disease have been found to be connected to the neuropsychiatric abnormalities frequently observed in Darier disease (Gordon-Smith et al., 2010). The CNV duplications on chromosome 16p13.1 have been shown to be a risk factor for neuropsychiatric disorders, including schizophrenia (Ingason et al., 2011), and the candidate genes within the region of this CNV duplication that are associated with schizophrenia include the NTAN1 and NDE1 genes, which encode nuclear N-terminal asparagine amidase 1 and distribution protein nude homolog 1 proteins, respectively. Further studies have shown that the NDE1 protein functions as the partner of DISC1 and thereby participates in the DISC1 pathway, which has been implicated in synaptic plasticity, neurotransmission, and neurodevelopment (Fullston et al., 2011; Ingason et al., 2011). The CNV deletion on chromosome 22q11.2 is among the first identified CNVs that is associated with an increased risk for schizophrenia. Further analysis displayed that important candidate genes within the intervals include the phosphatidyl-inositol-4-kinase-catalytic-alpha (PIK4CA) gene (Vorstman et al., 2009). The PIK4CA gene encodes the enzymatic protein in the phosphatidylinositol (PI) pathway (Vorstman et al., 2009), and it is thought that the PI pathway plays an important role in the modulation of synaptic transmission and signal transduction (Jungerius et al., 2008). Interestingly, the PIK4CA gene is well-documented to be highly expressed in the gray matter, with significantly higher levels in the fetal brain than in the adult brain (Nakagawa et al., 1996), suggesting an important role for PIK4CA in neurodevelopment and in neurodevelopment-related mental diseases such as schizophrenia; these findings are in agreement with the neurodevelopmental pathogenesis of schizophrenia (Bassett et al., 2001). More detailed information about the major pathogenic CNVs and affected genes identified to be associated with schizophrenia is summarized in Table 1. CNVs that have been demonstrated to disrupt the expression of single or multiple genes associated with schizophrenia account for more of the genetic burden of schizophrenia; however, it remains largely unexplored which genes contribute to the phenotypes. In addition, among the CNVs, the rare CNVs are more likely to exert a greater effect on individuals with schizophrenia, compared with unaffected persons, and on those with disease onset before the age of 18 years old. The lines of evidence have suggested that schizophrenia-related CNVs have been strongly associated with the alteration of genes involved in signal transmission between brain cells, and those genes hold promise as therapeutic targets for the development of molecular targeted agents of signal transmission in the treatment of schizophrenia.

**Table 1:** Major susceptibility CNVs for schizophrenia.

**Chromosomal locus**	**Type of CNV (deletions/duplications)**	**Disrupted genes/functions**	**References**
1q21.1	Deletions and Duplications	34 genes; intercellular exchanges of neurotransmitters such as glutamate in synaptic transmission of the central nervous system	International Schizophrenia, 2008; Mefford et al., 2008; Malhotra and Sebat, 2012
2p16.3	Deletions	1 gene *NRXN 1*; synthesis and release of neurotransmitters in synapses	International Schizophrenia, 2008; Kirov et al., 2008, 2009; Mefford et al., 2008; Sudhof, 2008; Walsh et al., 2008; Need et al., 2009; Ikeda et al., 2010; Malhotra and Sebat, 2012; Rosenfeld et al., 2012; Hamshere et al., 2013; Szatkiewicz et al., 2014
3q29	Deletions and Duplications	21 genes; synaptic plasticity, dendritic spine morphology, and glutamate transmission	Ni et al., 2007; Stefansson et al., 2008; Howard et al., 2010; Mulle et al., 2010; Nash et al., 2010; Rosenfeld et al., 2012; Yuan et al., 2014
7q11.23	Duplications	26–28 genes; anomalies of connective tissue and mental retardation	Ingason et al., 2011; Ripke et al., 2013
15q11.2	Deletions	4 genes; Mg^2+^ transportation	Zhou et al., 2008; Girirajan et al., 2011
15q11.2-13.1	Duplications	13–24 genes; glutamate neurotransmission	Malhotra and Sebat, 2012; Hamshere et al., 2013
15q13.3	Deletions	12 genes; mediation of rapid signal transmission at synapses	Zhao et al., 2013
16p11.2	Deletions and Duplications	29 genes; regulation of glucose homeostasis and muscular excitation and contraction	Nielsen et al., 2010; Mulle et al., 2014; Sinkus et al., 2015
16p13.1	Duplications	11 genes; synaptic plasticity, neurotransmission, and neurodevelopment through the DISC1 pathway	Gordon-Smith et al., 2010; Girirajan et al., 2011
22q11.2	Deletions and Duplications	84 genes; modulation of synaptic transmission and signal transduction through the phosphatidylinositol pathway	Nakagawa et al., 1996; Jungerius et al., 2008; Vorstman et al., 2009; Fullston et al., 2011

### Differences in risk genomic CNVs for schizophrenia have been noted among different populations

Geographic and ethnic diversities in the prevalence of schizophrenia have been well established, but the causes underlying the differences are not fully understood. Interestingly, differences in the large and rare schizophrenia-associated CNVs among populations have been reported (Szatkiewicz et al., 2014). In a study performed by Szatkiewicz and colleagues, a large sample size of subjects, including a total of 4719 schizophrenia patients and 5917 unaffected controls, were enrolled (Szatkiewicz et al., 2014). Aside from validating the elevated burden of the rare and large CNVs in schizophrenia and the significant correlation of the CNVs 16p11.2 dup, 22q11.2 del, and 3q29 del with this disease, the new CNV 17q12 dup in the Swedish population was found to be associated with schizophrenia; this CNV was previously implicated in autism spectrum disorders and mental retardation (Szatkiewicz et al., 2014). In a study of schizophrenia-associated CNVs in the Han Chinese population, Shi et al. compared a total of 155 schizophrenic individuals with 187 unaffected controls and found that there was no significant enrichment in rare CNVs greater than 100 kbp in length in the case group, compared to the control group (Shi et al., 2008). Similar results also have been obtained by a Japanese research group, who did not observe a significant increase in rare CNVs larger than 500 kbp (Ikeda et al., 2010). It is likely that distinct geographic environments that global populations could be exposed to may increase shifts in genetic factors such as CNVs, and evolution may also affect differences in CNVs. Since the CNVs that confer high risk for schizophrenia have been investigated mainly in the European and North American populations, even larger differences in CNVs among global populations may exist to influence the risk for schizophrenia. To better understand the alterations, including similarities and differences of CNVs in schizophrenia among populations worldwide, and characterize the global patterns of risk CNVs for schizophrenia, comparative studies with more geographically diverse populations from Asia, Africa, and South America should be carried out, thus allowing scientists to identify and characterize specific CNVs as markers to predict the development and early onset of schizophrenia in separate populations.

### Potential applications for genomic CNVs in schizophrenia

The new advanced genetic technologies have offered great opportunity for schizophrenia research to decipher the molecular mechanisms underlying the early onset and development of this disease as well as to provide potential clinical applications in genetic counseling, diagnosis, and therapy for schizophrenia.

### Potential value of manipulating genomic CNVs in schizophrenia research

Manipulation of the pathogenic CNVs significantly associated with schizophrenia will usher in a potentially novel direction for schizophrenia research. CNVs usually range from an intermediate size (more than 50 bp in length) to a large size (several Mbps) and occur mainly as deletions and duplications in the human genome. The pathogenic CNVs are typically rare in the population and large in size, which has raised huge challenges in the editing of CNVs. Fortunately, the new CRISPR/Cas9 genetic editing system has been recently developed and has received great attention (Doudna and Charpentier, 2014; Sander and Joung, 2014). The CRISPR/Cas9 genome editing system relies on two major components: the Cas9 protein and the guide RNA (gRNA) (Doudna and Charpentier, 2014). Directed by the gRNA that is complementary to the CRISPR RNA, Cas9, a protein with endonuclease activity, is able to reach the specified target site and subsequently cleave the genomic DNA. With a donor template containing the desired alterations, the CRISPR/Cas9 system enables researchers to manipulate the genomic DNA, including deletions or insertions in cells, plants, and entire animal models (Xiao et al., 2013; Hsu et al., 2014; Sander and Joung, 2014; Huang et al., 2015; Liang et al., 2015; Callaway, 2016; Long et al., 2016; Nelson et al., 2016; Paquet et al., 2016; Tabebordbar et al., 2016). Considerable progress in genomic DNA editing using the CRISPR/Cas9 genetic editing system has been made over the past few years. For example, with optimizations in the CRISPR/Cas9 genetic editing technology, Tai and colleagues have been able to generate successfully reciprocal CNVs of 16p11.2 (740-kb deletion) and 15q13.3 (2-Mb deletion) in human induced pluripotent stem cells (iPSCs) (Tai et al., 2016). Of note, no off-target CNVs were reported in this study and the approach was reproducible. The successful creation of reciprocal CNVs in iPSCs also has opened a possible direction toward introduction or correction of the rare and large pathogenic CNVs in cells and animal models for disease research including schizophrenia, which will allow us to manipulate pathogenic CNVs. Therefore, the CRIPR/Cas9 system could be used in animal and cell culture experiments to better understand how these genetic alterations affect gene expression or protein function and to advance our knowledge in terms of the pathological mechanisms underlying schizophrenia.

The application of the CRISPR/Cas9 genomic editing system in the reverse or introduction of the pathogenic CNVs for schizophrenia will potentially facilitate the generation of animal models for schizophrenia research as well. The lack of translatable animal model systems represents one of the major bottlenecks in schizophrenia research, leading to huge challenges not only in creating human disease symptoms in animal models but also tedious work and expensive costs for establishing animal models with specific genomic mutations, in particular, for the large CNVs using conventional approaches. Obviously, the CRISPR/Cas9 genomic editing system has provided a valuable approach to develop animal models for schizophrenia research concerning the pathogenic CNVs. Moreover, the CRISPR/Cas9 genetic tool has displayed a number of advantages in generating animal models with desired alterations in the genomic DNA. For instance, a conventional approach usually takes as long as 2 years to produce an animal model with the desired specific genetic alterations in the offspring, mainly because of the numerous breeding steps required. However, with the help of the CRISPR/Cas9 genetic editing system, it only takes up to 2 months to generate an animal model with the desired genomic alterations in the genomic DNA. Importantly, the CRISPR/Cas9 genetic editing system costs less than the traditional approach. Furthermore, with the optimized CRISPR/Cas9 system using multiple gRNAs instead of a single gRNA, this technology is able to create multiple desired mutations in the form of deletions or insertions in the embryo of animal models, whereas this is unlikely if other conventional methods are utilized. Thus, the application of the novel CRISPR/Cas9 genomic editing technology will likely speed up the pace of reasearch to better understand the CNVs in schizophrenia.

### Potential diagnostic value of CNVs to identify individuals at risk of developing schizophrenia or having early-onset schizophrenia

Our understanding of the roles for CNVs in schizophrenia is in its infancy, but the area is growing rapidly. It has become clearer that CNVs, as one form of genetic alterations, exert a profound effect on the schizophrenia-related genes harbored within the CNVs and that some of these changes may be sufficient to cause the early onset and development of schizophrenia. Carriers of these CNVs, in particular, if the alterations turn out to disrupt the expression of schizophrenia-associated genes, are likely to be at great risk of developing schizophrenia. In a recent study, Ahn and colleagues found that childhood onset schizophrenia subjects, who are defined as the disease onset earlier than the age of 13 years, exhibited significantly higher rate of schizophrenia-related CNVs than that in adult onset schizophrenia patients (Ahn et al., 2014). It is also worth attention to the first evidence that a large del CNV with 2.5–3 Mb in size at 22q11.2 has been identified to be more associated with childhood onset schizophrenia than adult onset schizophrenia. These novel findings suggest that early onset schizophrenia patients share CNV defects in the brain development, which may hold promise as potential markers to help predict or diagnose schizophrenia, especially the childhood-onset schizophrenia due to considerable challenges in the timely diagnosis (Ahn et al., 2014). Although the fact that schizophrenia-associated CNVs including those related to the childhood onset schizophrenia have been identified, however, the following critical challenging issues remain to be addressed in future: to which extent this is a pathogenic mechanism underlying the childhood onset schizophrenia, or the development of schizophrenia in typical population. More studies in larger population to measure the burdens of the disease-related CNVs in ways of the number of the CNVs and the number of the disease-related genes covered the CNVs are needed. In addition, some CNVs that have been found to be associated with schizophrenia and have been linked to a specific population are worthy of attention as well. Moreover, they may hold promise as genetic markers to identify those at risk of developing schizophrenia or as potential molecular markers of schizophrenia susceptibility and onset of schizophrenia in specific populations.

### Potential value of correction of the pathogenic genomic CNVs in schizophrenia therapy

Currently, medications along with psychosocial therapy are well-accepted approaches for the care of patients suffering from schizophrenia. However, a large proportion of schizophrenia patients fail to comply with the prescribed medications, mainly due to their serious side effects, thus resulting in a large number of schizophrenia cases going untreated. However, it has been considered that non-adherence of medications may be a common problem in every branch of medicine, and is often more serious among patients with mental disorders including schizophrenia, provided that mental disorders may impair patients' decision-making and their capacity to recognize the severity of the illness (Pompili et al., 2013). As CNVs are one of the major forms of genetic variations involved in the pathogenesis of schizophrenia, correction of the pathogenic genes with the CRISPR/Cas9 system may become available. Indeed, genomic alterations of a large size like CNVs have successfully replaced mutations with the correct form at the genomic DNA level in both cells and animal models. Recently, with the help of CRISPR/Cas9 genetic editing technology, a research team has successfully manipulated large-size reciprocal CNVs (16p11.2, 740-kb deletion and 15q13.3, 2-Mb deletion) in human iPSCs and entire animals without observing off-target CNVs. In addition, it has been reported recently that CRISPR/Cas9 gene editing technology has empowered scientists to successfully treat human disease in an entire animal model (Nelson et al., 2016). These results show great potential, and the technology has brought new hope for the genetic therapy of many other genetically related diseases like schizophrenia.

## Challenges and future directions

Although rapid progress has been made in our understating of CNVs in schizophrenia, this emerging area requires further exploration in the future. First, given the increasing correlation of CNVs with schizophrenia, there is a need to study the large and rare CNVs as an important genetic component in the disease. Future genetic studies of schizophrenia will need to take CNVs into account. Otherwise, important genetic information related to disease development may be missing. Second, schizophrenia is a complex genetic disease and multiple components of heritability have been thought to be involved in the etiology of this disease. There is also a call for broad investigations into additional genetic factors for schizophrenia, such as combining CNVs with other forms of genetic variations like SNPs in an effort to establish a more complex model of schizophrenia research. Third, it has become more apparent that the CNVs themselves, even if in combination with other genetic factors, may not be sufficient to develop schizophrenia. Through interacting with environmental factors, they may trigger the early onset and development of schizophrenia. These factors have largely made studying the molecular etiology of schizophrenia even more complex and challenging. Fourth, the discovery of population- or ethnicity-specific CNVs associated with schizophrenia will need a large sample size of well-diagnosed schizophrenia patients and matched unaffected control individuals. This study will require the recruitment and comparative studies of several thousands of cases with broader populations and ethnicities across the world, since the majority of investigations conducted to date have focused on European and North American populations. The difficulties presented cannot be overstated. Finally, it will be challenging to characterize which CNVs are linked to behavioral and neuropsychiatric phenotypes before proper animal models are established.

In addition, challenges may arise from the immature techniques of the CRISPR/Cas9 genomic editing system in the correction of defects in the pathogenic CNVs. Several challenging issues related to the CRISPR/Cas9 technique, including off-target cleavage by the Cas9 protein, and difficulty in delivering the CRISPR/Cas9 components across the blood–brain barrier, will need to be improved in future. Furthermore, the schizophrenia-associated CNVs are generally large in size, which has posed additional challenges in trying to reverse the defect CNVs with the CRISPR/Cas9 system. Fortunately, it has been reported recently that a number of genetically engineered viruses have become available to carry the CRISPR/Cas9 components, pass through the blood–brain barrier, and finally deliver those components to brain cells without the observation of causative diseases (Deverman et al., 2016). Meanwhile, ethical concerns about the application of CRISPR/Cas9 technology in human diseases using embryos remain to be resolved (Krishan et al., 2016). It seems that there is still a long way to go before the CRISPR/Cas9 genetic approach can be applied to correct genetic defects, including the pathogenic CNVs for the treatment of schizophrenia.

## Author contributions

CZ: conceptual design and writing of the draft manuscript; WH: conceptual design and writing of the final manuscript; CL, LH, and JL collected and examined the enrolled articles in this review.

### Conflict of interest statement

The authors declare that the research was conducted in the absence of any commercial or financial relationships that could be construed as a potential conflict of interest.

## References

[B1] Ahn K. Gotay N. Andersen T. M. Anvari A. A. Gochman P. Lee Y.. (2014). High rate of disease-related copy number variations in childhood onset schizophrenia. Mol. Psychiatry 19, 568–572. 10.1038/mp.2013.59 23689535 PMC5157161

[B2] Bassett A. S. Chow E. W. O'Neill S. Brzustowicz L. M. (2001). Genetic insights into the neurodevelopmental hypothesis of schizophrenia. Schizophr. Bull. 27, 417–430. 10.1093/oxfordjournals.schbul.a006884 11596844

[B3] Callaway E. (2016). Gene-editing research in human embryos gains momentum. Nature 532, 289–290. 10.1038/532289a 27111607

[B4] Cardno A. G. Marshall E. J. Coid B. Macdonald A. M. Ribchester T. R. Davies N. J.. (1999). Heritability estimates for psychotic disorders: the Maudsley twin psychosis series. Arch. Gen. Psychiatry 56, 162–168. 10.1001/archpsyc.56.2.162 10025441

[B5] Chen J. Cao F. Liu L. Wang L. Chen X. (2015). Genetic studies of schizophrenia: an update. Neurosci. Bull. 31, 87–98. 10.1007/s12264-014-1494-4 25652814 PMC5562645

[B6] Crow T. J. (1999). Schizophrenia as an epigenetic puzzle-in what sense were Gottesman and Shields correct? Mol. Psychiatry 4:S18.

[B7] Deverman B. E. Pravdo P. L. Simpson B. P. Kumar S. R. Chan K. Y. Banerjee A.. (2016). Cre-dependent selection yields AAV variants for widespread gene transfer to the adult brain. Nat. Biotechnol. 34, 204–209. 10.1038/nbt.3440 26829320 PMC5088052

[B8] Doudna J. A. Charpentier E. (2014). Genome editing. The new frontier of genome engineering with CRISPR-Cas9. Science 346:1258096. 10.1126/science.1258096 25430774

[B9] Foster A. Gable J. Buckley J. (2012). Homelessness in schizophrenia. Psychiatr. Clin. North Am. 35, 717–734. 10.1016/j.psc.2012.06.010 22929875

[B10] Fullston T. Gabb B. Callen D. Ullmann R. Woollatt E. Bain S.. (2011). Inherited balanced translocation t(9;17)(q33.2;q25.3) concomitant with a 16p13.1 duplication in a patient with schizophrenia. Am. J. Med. Genet. B Neuropsychiatr. Genet. 156, 204–214. 10.1002/ajmg.b.31157 21302349

[B11] Girirajan S. Campbell C. D. Eichler E. E. (2011). Human copy number variation and complex genetic disease. Annu. Rev. Genet. 45, 203–226. 10.1146/annurev-genet-102209-163544 21854229 PMC6662611

[B12] Global Burden of Disease Study C. (2015). Global, regional, and national incidence, prevalence, and years lived with disability for 301 acute and chronic diseases and injuries in 188 countries, 1990-2013: a systematic analysis for the Global Burden of Disease Study 2013. Lancet 386, 743–800. 10.1016/S0140-6736(15)60692-4 26063472 PMC4561509

[B13] Gordon-Smith K. Jones L. A. Burge S. M. Munro C. S. Tavadia S. Craddock N. (2010). The neuropsychiatric phenotype in Darier disease. Br. J. Dermatol. 163, 515–522. 10.1111/j.1365-2133.2010.09834.x 20456342

[B14] Grayton H. M. Fernandes C. Rujescu D. Collier D. A. (2012). Copy number variations in neurodevelopmental disorders. Prog. Neurobiol. 99, 81–91. 10.1016/j.pneurobio.2012.07.005 22813947

[B15] Guha S. Rees E. Darvasi A. Ivanov D. Ikeda M. Bergen S. E.. (2013). Implication of a rare deletion at distal 16p11.2 in schizophrenia. JAMA Psychiatry 70, 253–260. 10.1001/2013.jamapsychiatry.71 23325106 PMC3750982

[B16] Hamshere M. L. Walters J. T. Smith R. Richards A. L. Green E. Grozeva D.. (2013). Genome-wide significant associations in schizophrenia to ITIH3/4, CACNA1C and SDCCAG8, and extensive replication of associations reported by the Schizophrenia, PGC. Mol. Psychiatry 18, 708–712. 10.1038/mp.2012.67 22614287 PMC4724864

[B17] Hor K. Taylor M. (2010). Suicide and schizophrenia: a systematic review of rates and risk factors. J. Psychopharmacol. 24, 81–90. 10.1177/1359786810385490 20923923 PMC2951591

[B18] Howard M. A. Elias G. M. Elias L. A. Swat W. Nicoll R. A. (2010). The role of SAP97 in synaptic glutamate receptor dynamics. Proc. Natl. Acad. Sci. U.S.A. 107, 3805–3810. 10.1073/pnas.0914422107 20133708 PMC2840522

[B19] Hsu P. D. Lander E. S. Zhang F. (2014). Development and applications of CRISPR-Cas9 for genome engineering. Cell 157, 1262–1278. 10.1016/j.cell.2014.05.010 24906146 PMC4343198

[B20] Huang X. Wang Y. Yan W. Smith C. Ye Z. Wang J.. (2015). Production of gene-corrected adult beta globin protein in human erythrocytes differentiated from patient iPSCs after genome editing of the sickle point mutation. Stem Cells 33, 1470–1479. 10.1002/stem.1969 25702619 PMC4628786

[B21] Ikeda M. Aleksic B. Kirov G. Kinoshita Y. Yamanouchi Y. Kitajima T.. (2010). Copy number variation in schizophrenia in the Japanese population. Biol. Psychiatry 67, 283–286. 10.1016/j.biopsych.2009.08.034 19880096

[B22] Ingason A. Rujescu D. Cichon S. Sigurdsson E. Sigmundsson T. Pietilainen O. P.. (2011). Copy number variations of chromosome 16p13.1 region associated with schizophrenia. Mol. Psychiatry 16, 17–25. 10.1038/mp.2009.101 19786961 PMC3330746

[B23] International Schizophrenia C. (2008). Rare chromosomal deletions and duplications increase risk of schizophrenia. Nature 455, 237–241. 10.1038/nature07239 18668038 PMC3912847

[B24] International Schizophrenia C. Purcell S. M. Wray N. R. Stone J. L. Visscher P. M. O'Donovan M. C.. (2009). Common polygenic variation contributes to risk of schizophrenia and bipolar disorder. Nature 460, 748–752. 10.1038/nature08185 19571811 PMC3912837

[B25] Jungerius B. J. Hoogendoorn M. L. Bakker S. C. Van't Slot R. Bardoel A. F. Ophoff R. A.. (2008). An association screen of myelin-related genes implicates the chromosome 22q11 PIK4CA gene in schizophrenia. Mol. Psychiatry 13, 1060–1068. 10.1038/sj.mp.4002080 17893707

[B26] Kavanagh D. H. Tansey K. E. O'Donovan M. C. Owen M. J. (2015). Schizophrenia genetics: emerging themes for a complex disorder. Mol. Psychiatry 20, 72–76. 10.1038/mp.2014.148 25385368

[B27] Kirov G. Gumus D. Chen W. Norton N. Georgieva L. Sari M.. (2008). Comparative genome hybridization suggests a role for NRXN1 and APBA2 in schizophrenia. Hum. Mol. Genet. 17, 458–465. 10.1093/hmg/ddm323 17989066

[B28] Kirov G. Pocklington A. J. Holmans P. Ivanov D. Ikeda M. Ruderfer D.. (2012). De novo CNV analysis implicates specific abnormalities of postsynaptic signalling complexes in the pathogenesis of schizophrenia. Mol. Psychiatry 17, 142–153. 10.1038/mp.2011.154 22083728 PMC3603134

[B29] Kirov G. Rujescu D. Ingason A. Collier D. A. O'Donovan M. C. Owen M. J. (2009). Neurexin 1 (NRXN1) deletions in schizophrenia. Schizophr. Bull. 35, 851–854. 10.1093/schbul/sbp079 19675094 PMC2728827

[B30] Krishan K. Kanchan T. Singh B. (2016). Human genome editing and ethical considerations. Sci. Eng. Ethics 22, 597–599. 10.1007/s11948-015-9675-8 26154417

[B31] Levinson D. F. Duan J. Oh S. Wang K. Sanders A. R. Shi J.. (2011). Copy number variants in schizophrenia: confirmation of five previous findings and new evidence for 3q29 microdeletions and VIPR2 duplications. Am. J. Psychiatry 168, 302–316. 10.1176/appi.ajp.2010.10060876 21285140 PMC4441324

[B32] Liang P. Xu Y. Zhang X. Ding C. Huang R. Zhang Z.. (2015). CRISPR/Cas9-mediated gene editing in human tripronuclear zygotes. Protein Cell 6, 363–372. 10.1007/s13238-015-0153-5 25894090 PMC4417674

[B33] Long C. Amoasii L. Mireault A. A. McAnally J. R. Li H. Sanchez-Ortiz E.. (2016). Postnatal genome editing partially restores dystrophin expression in a mouse model of muscular dystrophy. Science 351, 400–403. 10.1126/science.aad5725 26721683 PMC4760628

[B34] Malhotra D. Sebat J. (2012). CNVs: harbingers of a rare variant revolution in psychiatric genetics. Cell 148, 1223–1241. 10.1016/j.cell.2012.02.039 22424231 PMC3351385

[B35] Mefford H. C. Sharp A. J. Baker C. Itsara A. Jiang Z. Buysse K.. (2008). Recurrent rearrangements of chromosome 1q21.1 and variable pediatric phenotypes. N. Engl. J. Med. 359, 1685–1699. 10.1056/NEJMoa0805384 18784092 PMC2703742

[B36] Moreno-De-Luca D. Sanders S. J. Willsey A. J. Mulle J. G. Lowe J. K. Geschwind D. H.. (2013). Using large clinical data sets to infer pathogenicity for rare copy number variants in autism cohorts. Mol. Psychiatry 18, 1090–1095. 10.1038/mp.2012.138 23044707 PMC3720840

[B37] Mulle J. G. Dodd A. F. McGrath J. A. Wolyniec P. S. Mitchell A. A. Shetty A. C.. (2010). Microdeletions of 3q29 confer high risk for schizophrenia. Am. J. Hum. Genet. 87, 229–236. 10.1016/j.ajhg.2010.07.013 20691406 PMC2917706

[B38] Mulle J. G. Pulver A. E. McGrath J. A. Wolyniec P. S. Dodd A. F. Cutler D. J.. (2014). Reciprocal duplication of the Williams-Beuren syndrome deletion on chromosome 7q11.23 is associated with schizophrenia. Biol. Psychiatry 75, 371–377. 10.1016/j.biopsych.2013.05.040 23871472 PMC3838485

[B39] Nakagawa T. Goto K. Kondo H. (1996). Cloning, expression, and localization of 230-kDa phosphatidylinositol 4-kinase. J. Biol. Chem. 271, 12088–12094. 10.1074/jbc.271.20.12088 8662589

[B40] Nash J. E. Appleby V. J. Correa S. A. Wu H. Fitzjohn S. M. Garner C. C.. (2010). Disruption of the interaction between myosin VI and SAP97 is associated with a reduction in the number of AMPARs at hippocampal synapses. J. Neurochem. 112, 677–690. 10.1111/j.1471-4159.2009.06480.x 19895665

[B41] Need A. C. Ge D. Weale M. E. Maia J. Feng S. Heinzen E. L.. (2009). A genome-wide investigation of SNPs and CNVs in schizophrenia. PLoS Genet. 5:e1000373. 10.1371/annotation/e0196ebb-de40-453f-8f8c-791b126618da 19197363 PMC2631150

[B42] Nelson C. E. Hakim C. H. Ousterout D. G. Thakore P. I. Moreb E. A. Castellanos Rivera R. M.. (2016). *In vivo* genome editing improves muscle function in a mouse model of Duchenne muscular dystrophy. Science 351, 403–407. 10.1126/science.aad5143 26721684 PMC4883596

[B43] Ni X. Valente J. Azevedo M. H. Pato M. T. Pato C. N. Kennedy J. L. (2007). Connexin 50 gene on human chromosome 1q21 is associated with schizophrenia in matched case control and family-based studies. J. Med. Genet. 44, 532–536. 10.1136/jmg.2006.047944 17412882 PMC2597930

[B44] Nielsen J. Dahm M. Lublin H. Taylor D. (2010). Psychiatrists' attitude towards and knowledge of clozapine treatment. J. Psychopharmacol. 24, 965–971. 10.1177/0269881108100320 19164499

[B45] Owen M. J. Sawa A. Mortensen P. B. (2016). Schizophrenia. Lancet 388, 86–97. 10.1016/S0140-6736(15)01121-6 26777917 PMC4940219

[B46] Paquet D. Kwart D. Chen A. Sproul A. Jacob S. Teo S.. (2016). Efficient introduction of specific homozygous and heterozygous mutations using CRISPR/Cas9. Nature 533, 125–129. 10.1038/nature17664 27120160

[B47] Pompili M. Venturini P. Palermo M. Stefani H. Seretti M. E. Lamis D. A.. (2013). Mood disorders medications: predictors of nonadherence - review of the current literature. Expert Rev. Neurother. 13, 809–825. 10.1586/14737175.2013.811976 23898852

[B48] Purcell S. M. Moran J. L. Fromer M. Ruderfer D. Solovieff N. Roussos P.. (2014). A polygenic burden of rare disruptive mutations in schizophrenia. Nature 506, 185–190. 10.1038/nature12975 24463508 PMC4136494

[B49] Ripke S. O'Dushlaine C. Chambert K. Moran J. L. Kahler A. K. Akterin S.. (2013). Genome-wide association analysis identifies 13 new risk loci for schizophrenia. Nat. Genet. 45, 1150–1159. 10.1038/ng.2742 23974872 PMC3827979

[B50] Rosenfeld J. A. Traylor R. N. Schaefer G. B. McPherson E. W. Ballif B. C. Klopocki E.. (2012). Proximal microdeletions and microduplications of 1q21.1 contribute to variable abnormal phenotypes. Eur. J. Hum. Genet. 20, 754–761. 10.1038/ejhg.2012.6 22317977 PMC3376272

[B51] Sander J. D. Joung J. K. (2014). CRISPR-Cas systems for editing, regulating and targeting genomes. Nat. Biotechnol. 32, 347–355. 10.1038/nbt.2842 24584096 PMC4022601

[B52] Shi Y. Y. He G. Zhang Z. Tang W. Zhang J. Jr. Zhao Q.. (2008). A study of rare structural variants in schizophrenia patients and normal controls from Chinese Han population. Mol. Psychiatry 13, 911–913. 10.1038/mp.2008.69 18800052

[B53] Sinkus M. L. Graw S. Freedman R. Ross R. G. Lester H. A. Leonard S. (2015). The human CHRNA7 and CHRFAM7A genes: a review of the genetics, regulation, and function. Neuropharmacology 96, 274–288. 10.1016/j.neuropharm.2015.02.006 25701707 PMC4486515

[B54] St Clair D. (2009). Copy number variation and schizophrenia. Schizophr. Bull. 35, 9–12. 10.1093/schbul/sbn147 18990708 PMC2643970

[B55] Stefansson H. Rujescu D. Cichon S. Pietilainen O. P. Ingason A. Steinberg S.. (2008). Large recurrent microdeletions associated with schizophrenia. Nature 455, 232–236. 10.1038/nature07229 18668039 PMC2687075

[B56] Sudhof T. C. (2008). Neuroligins and neurexins link synaptic function to cognitive disease. Nature 455, 903–911. 10.1038/nature07456 18923512 PMC2673233

[B57] Szatkiewicz J. P. O'Dushlaine C. Chen G. Chambert K. Moran J. L. Neale B. M.. (2014). Copy number variation in schizophrenia in Sweden. Mol. Psychiatry 19, 762–773. 10.1038/mp.2014.40 24776740 PMC4271733

[B58] Tabebordbar M. Zhu K. Cheng J. K. Chew W. L. Widrick J. J. Yan W. X.. (2016). *In vivo* gene editing in dystrophic mouse muscle and muscle stem cells. Science 351, 407–411. 10.1126/science.aad5177 26721686 PMC4924477

[B59] Tai D. J. Ragavendran A. Manavalan P. Stortchevoi A. Seabra C. M. Erdin S.. (2016). Engineering microdeletions and microduplications by targeting segmental duplications with CRISPR. Nat. Neurosci. 19, 517–522. 10.1038/nn.4235 26829649 PMC4903018

[B60] Vorstman J. A. Chow E. W. Ophoff R. A. van Engeland H. Beemer F. A. Kahn R. S.. (2009). Association of the PIK4CA schizophrenia-susceptibility gene in adults with the 22q11.2 deletion syndrome. Am. J. Med. Genet. B Neuropsychiatr. Genet. 150B, 430–433. 10.1002/ajmg.b.30827 18646052 PMC3127866

[B61] Walsh T. McClellan J. M. McCarthy S. E. Addington A. M. Pierce S. B. Cooper G. M.. (2008). Rare structural variants disrupt multiple genes in neurodevelopmental pathways in schizophrenia. Science 320, 539–543. 10.1126/science.1155174 18369103

[B62] Xiao A. Wang Z. Hu Y. Wu Y. Luo Z. Yang Z.. (2013). Chromosomal deletions and inversions mediated by TALENs and CRISPR/Cas in zebrafish. Nucleic Acids Res. 41, e141. 10.1093/nar/gkt464 23748566 PMC3737551

[B63] Yuan J. Jin C. Sha W. Zhou Z. Zhang F. Wang M.. (2014). A competitive PCR assay confirms the association of a copy number variation in the VIPR2 gene with schizophrenia in Han Chinese. Schizophr. Res. 156, 66–70. 10.1016/j.schres.2014.04.004 24794882

[B64] Zhao Q. Li T. Zhao X. Huang K. Wang T. Li Z.. (2013). Rare CNVs and tag SNPs at 15q11.2 are associated with schizophrenia in the Han Chinese population. Schizophr. Bull. 39, 712–719. 10.1093/schbul/sbr197 22317777 PMC3627771

[B65] Zhou W. Zhang L. Guoxiang X. Mojsilovic-Petrovic J. Takamaya K. Sattler R.. (2008). GluR1 controls dendrite growth through its binding partner, SAP97. J. Neurosci. 28, 10220–10233. 10.1523/JNEUROSCI.3434-08.2008 18842882 PMC2699678

